# Assessment of Seroprevalence of SARS-CoV-2 and Risk Factors Associated With COVID-19 Infection Among Outpatients in Virginia

**DOI:** 10.1001/jamanetworkopen.2020.35234

**Published:** 2021-02-08

**Authors:** Elizabeth T. Rogawski McQuade, Kristin A. Guertin, Lea Becker, Darwin Operario, Jean Gratz, Dave Guan, Fauzia Khan, Jennifer White, Timothy L. McMurry, Bhruga Shah, Stephanie Garofalo, Matt Southerland, Kelly Bear, John Brush, Cynthia Allen, Amy Frayser, Rebecca Vokes, Rashmi Pershad, Lilian Peake, Christopher deFilippi, Kathleen Barackman, Gonzalo Bearman, Andrea Bidanset, Francis Farrell, David Trump, Eric R. Houpt

**Affiliations:** 1 Division of Infectious Diseases and International Health, University of Virginia, Charlottesville; 2 Department of Public Health Sciences, University of Virginia, Charlottesville; 3 Research and Clinical Trial Analytics Team, Division of Quality Performance Improvement, University of Virginia, Charlottesville; 4 Inova Heart and Vascular Institute, Inova Health System, Falls Church, Virginia; 5 Office of Clinical Research, Sentara Healthcare, Norfolk, Virginia; 6 Division of Infectious Diseases, Virginia Commonwealth University, Richmond; 7 Virginia Department of Health, Richmond; 8 Carilion Clinic, Roanoke, Virginia

## Abstract

**Importance:**

Data from seroepidemiologic surveys measuring severe acute respiratory syndrome coronavirus 2 (SARS-CoV-2) exposure in diverse communities and ascertaining risk factors associated with infection are important to guide future prevention strategies.

**Objective:**

To assess the prevalence of previous SARS-CoV-2 infection across Virginia and the risk factors associated with infection after the first wave of coronavirus disease 2019 (COVID-19) infections in the US.

**Design, Setting, and Participants:**

In this statewide cross-sectional surveillance study, 4675 adult outpatients presenting for health care not associated with COVID-19 in Virginia between June 1 and August 14, 2020, were recruited to participate in a questionnaire and receive venipuncture to assess SARS-CoV-2 serology. Eligibility was stratified to meet age, race, and ethnicity quotas that matched regional demographic profiles.

**Main Outcomes and Measures:**

The main outcome was SARS-CoV-2 seropositivity, as measured by the Abbott SARS-CoV-2 immunoglobulin G assay.

**Results:**

Among 4675 adult outpatients (mean [SD] age, 48.8 [16.9] years; 3119 women [66.7%]; 3098 White [66.3%] and 4279 non-Hispanic [91.5%] individuals) presenting for non–COVID-19–associated health care across Virginia, the weighted seroprevalence was 2.4% (95% CI, 1.8%-3.1%) and ranged from 0% to 20% by zip code. Seroprevalence was notably higher among participants who were Hispanic (10.2%; 95% CI, 6.1%-14.3%), residing in the northern region (4.4%; 95% CI, 2.8%-6.1%), aged 40 to 49 years (4.4%; 95% CI, 1.8%-7.1%), and uninsured (5.9%; 95% CI, 1.5%-10.3%). Higher seroprevalence was associated with Hispanic ethnicity (adjusted odds ratio [aOR], 3.56; 95% CI, 1.76-7.21), residence in a multifamily unit (aOR, 2.55; 95% CI, 1.25-5.22), and contact with an individual with confirmed COVID-19 infection (aOR, 4.33; 95% CI, 1.77-10.58). The sensitivity of serology results was 94% (95% CI, 70%-100%) among those who reported receiving a previous polymerase chain reaction test for COVID-19 infection. Among 101 participants with seropositive results, 67 individuals (66.3%) were estimated to have asymptomatic infection. These data suggested a total estimated COVID-19 burden that was 2.8-fold higher than that ascertained by PCR-positive case counts.

**Conclusions and Relevance:**

This large statewide serologic study estimated that 2.4% of adults in Virginia had exposure to SARS-CoV-2, which was 2.8-fold higher than confirmed case counts. Hispanic ethnicity, residence in a multifamily unit, and contact with an individual with confirmed COVID-19 infection were significant risk factors associated with exposure. Most infections were asymptomatic. As of August 2020, the population in Virginia remained largely immunologically naive to the virus.

## Key Points

**Question:**

What percentage of the Virginia population had been exposed to severe acute respiratory syndrome coronavirus 2 after the first wave of coronavirus disease 2019 (COVID-19) infections in the US?

**Findings:**

In this cross-sectional study of 4675 adult outpatients presenting for non–COVID-19–associated health care in Virginia, a seroprevalence of approximately 2% was found, with an estimated 66% of seropositive results associated with asymptomatic infections. Hispanic ethnicity, residence in a multifamily unit, and contact with an individual with confirmed COVID-19 infection were risk factors significantly associated with exposure.

**Meaning:**

This study found that, as of August 2020, the population of Virginia remained largely immunologically naive to the virus.

## Teaser

This cross-sectional study assesses the statewide prevalence of previous infection with severe acute respiratory syndrome coronavirus 2 and the risk factors associated with infection among adult outpatients in Virginia.

## Introduction

The coronavirus disease 2019 (COVID-19) pandemic, caused by severe acute respiratory syndrome coronavirus 2 (SARS-CoV-2), has disrupted the US. Government agencies, health care systems, and the general public track confirmed case counts in US states to guide decision-making. Most of the confirmed case counts have been based on positive viral detection on the reverse transcriptase–polymerase chain reaction (PCR) test among individuals with symptoms. These case counts are underestimates of the total burden of SARS-CoV-2 infection because of incomplete testing availability and the substantial fraction of asymptomatic individuals with SARS-CoV-2 infection who never receive testing. Therefore, antibody evidence of previous SARS-CoV-2 exposure can be used as a complementary tool to measure the total burden of infection in the population.

There was initial concern that serologic assays would be subject to a high rate of false-positive results (ie, low specificity) because of exposure to other cross-reacting coronaviruses. False-negative results (ie, low sensitivity) were also a concern given the time required for the immune response to generate antibodies and the potential for dampened immune responses with asymptomatic infection. A number of studies, however, have indicated that the performance of many serologic assays is reasonable with regard to sensitivity and specificity. For example, the Abbott SARS-CoV-2 immunoglobulin G assay test has indicated 92.7% to 100% sensitivity and 99.4% to 100% specificity^1,2,3^ against a criterion standard of PCR-confirmed infection 14 days after symptom onset. Although sensitivity may be lower among asymptomatic individuals (78% sensitivity was reported in 1 study^3^), data regarding this population are incomplete.

Several serologic studies have been performed in the US to date. These studies have included large multiregional seroprevalence analyses that use residual clinical specimens from convenience samples^4^; recruitment of self-selected individuals within a particular geographic region^5,6^; surveys of high-risk individuals, such as first responders or health care workers; and community-level seroprevalence data from randomized households.^7,8^ Each strategy has limitations, such as generalizability to the public when participants are self-selected, inability to ascertain risk factors when no questionnaire is administered, and variable geographic reach and sample size constraints depending on the study design.

We conducted a statewide serologic survey of Virginia to examine the commonwealth’s total burden of previous COVID-19 infection. We enrolled participants at outpatient clinics or outpatient medical laboratories in 5 geographically disparate regions. To improve generalizability, we stratified enrollment to meet the age, race, and ethnicity profile of each region and did not include self-referred participants.

## Methods

### Study Design

The study was approved by the institutional review board of the University of Virginia, with a waiver of informed consent because the study did not constitute human subject research and because the study was requested by the Virginia Department of Health and therefore considered public health surveillance according to 45 CFR §46.102. This study followed the Strengthening the Reporting of Observational Studies in Epidemiology (STROBE) reporting guideline for cross-sectional studies.

Adults 18 years or older who presented in person for scheduled outpatient clinic or outpatient laboratory appointments were eligible. All outpatient sites conducted prescreening of individuals to ensure no COVID-19–like symptoms; at all sites, patients had options to convert in-person visits to telehealth visits. Enrollment occurred from June 1 to August 14, 2020, at 5 geographically diverse health system sites: the University of Virginia Health System in the northwest, INOVA Health System in the north, Sentara Healthcare in the east, Carilion Clinic in the southwest, and Virginia Commonwealth University in the central region. Each site enrolled up to 1000 Virginia residents, with up to 1 resident per household, and excluded individuals who were being evaluated for active COVID-19 illness. To improve generalizability, we stratified enrollment to meet the age, racial, and ethnic demographic profile of the region. Clinic sites were chosen to obtain this diversity. Population estimates for age, race, and ethnicity were obtained from the University of Virginia Weldon Cooper Center for Public Service based on the population as of July 1, 2019^9^ (population by zip code is shown in eFigure 1 in the Supplement). Race and ethnicity were defined by participants. If an individual’s characteristics aligned with the region’s demographic quota and the individual consented to participate in the study, research coordinators administered an electronic questionnaire and obtained a blood sample via venipuncture for SARS-CoV-2 serologic testing. Electronic informed consent was obtained from each participant.

All blood samples were tested at a central laboratory (University of Virginia Medical Laboratories, Charlottesville). Plasma (in lithium heparin tubes) was tested on the Architect i2000 analyzer (Abbott) using the SARS-CoV-2 immunoglobulin G antibody immunoassay approved via emergency use authorization. Participants were provided with their serology test results by mail.

### Statistical Analysis

All analyses were conducted using R software, version 3.6.3 (R Foundation for Statistical Computing). Sociodemographic characteristics were tabulated by region. To estimate the prevalence of seropositivity that was representative of the regional distributions of age, sex, race, ethnicity, and insurance status, we used an iterative proportional fitting procedure (also termed raking) to estimate sampling weights. Age was categorized into 18 to 29 years, 30 to 39 years, 40 to 49 years, 50 to 59 years, 60 to 69 years, 70 to 79 years, and 80 years or older. Race was defined according to census definitions of White, Black or African American, Asian, other race (specific races not queried on survey), and 2 or more races. Health insurance on January 1, 2020, was categorized as Medicaid, Medicare, private, military, and none or uninsured. Virginia population estimates by age, sex, race, and ethnicity at the regional level as of July 1, 2019, were collected.^9^ Virginia population estimates by insurance status at the regional level were collected from 2018 US Census Bureau data.^10^ The raked weights were estimated using the R software survey package^11^ to match the cross-classified distribution of age and sex and the marginal distribution of race, ethnicity, and health insurance at the regional level.

The raw prevalence of SARS-CoV-2 seropositivity by region and subgroup was estimated as the proportion of samples with positive results. The weighted seropositivity prevalence was estimated, accounting for the estimated raked weights using the R survey package. Prevalence was further corrected for imperfect sensitivity and specificity of the serology assay using the formula:

*P_corrected_* = (*P* + *Sp* − 1)/(*Se* + *Sp* − 1),

where *P* is the weighted prevalence, *Se* is the sensitivity of the assay, and *Sp* is the specificity of the assay. Sensitivity and specificity were assumed to be 92.7% (95% CI, 90.2%-94.8%) and 99.9% (95% CI, 99.4%-100%), respectively.^3^ Variability around these estimates was incorporated into the calculation of 95% CIs for the corrected prevalence rates using the delta method. Subgroups categorized by age, sex, race, ethnicity, insurance status, high-risk health condition, and week of collection were assessed.

To estimate rates of underascertainment, we estimated the expected total number of SARS-CoV-2 infections per region by multiplying the prevalence by the regional population of individuals 18 years and older. We compared this number to the number of cases per region among individuals 18 years and older, excluding cases among adults living in institutional facilities (long-term care, correctional, or congregate facilities), as reported by the Virginia Department of Health. We calculated the ratio of estimated infections to reported cases with ranges derived from the 95% CIs of the seroprevalence estimates. We identified risk factors associated with SARS-CoV-2 seropositivity using logistic regression analysis. We assessed region, sociodemographic characteristics, living environment, working environment, contact with someone with confirmed COVID-19 infection, perception of risk, perception of adherence to prevention behaviors, and travel since January 1, 2020. Risk factors with univariable odds ratios (ORs) that were statistically significant (*P* < .05) or univariable ORs that were less than 0.67 or greater than 1.50 were included in the multivariable model.

## Results

A total of 6523 individuals were approached for study participation, 5061 individuals were interested in participating, and 4841 individuals were eligible to participate. Of those who were eligible, 4675 participants (96.5%; mean [SD] age, 48.8 [16.9] years; 3119 women [66.7%]; 3098 White [66.3%] and 4279 non-Hispanic [91.5%] individuals) enrolled and had complete demographic questionnaire responses and blood test results available. Participants were enrolled at 5 large health systems located in 5 regions of the state: central (Virginia Commonwealth University; n = 865), east (Sentara Healthcare; n = 976), north (INOVA Health System; n = 1001), northwest (University of Virginia Health System; n = 994), and southwest (Carilion Clinic; n = 839). Although participant residence was concentrated around these 5 centers, there was substantial geographic representation across the state, with 475 of 873 residential zip codes (54.4%) represented (Figure). The complete characteristics of the study population are shown in the eTable in the Supplement.

**Figure.**
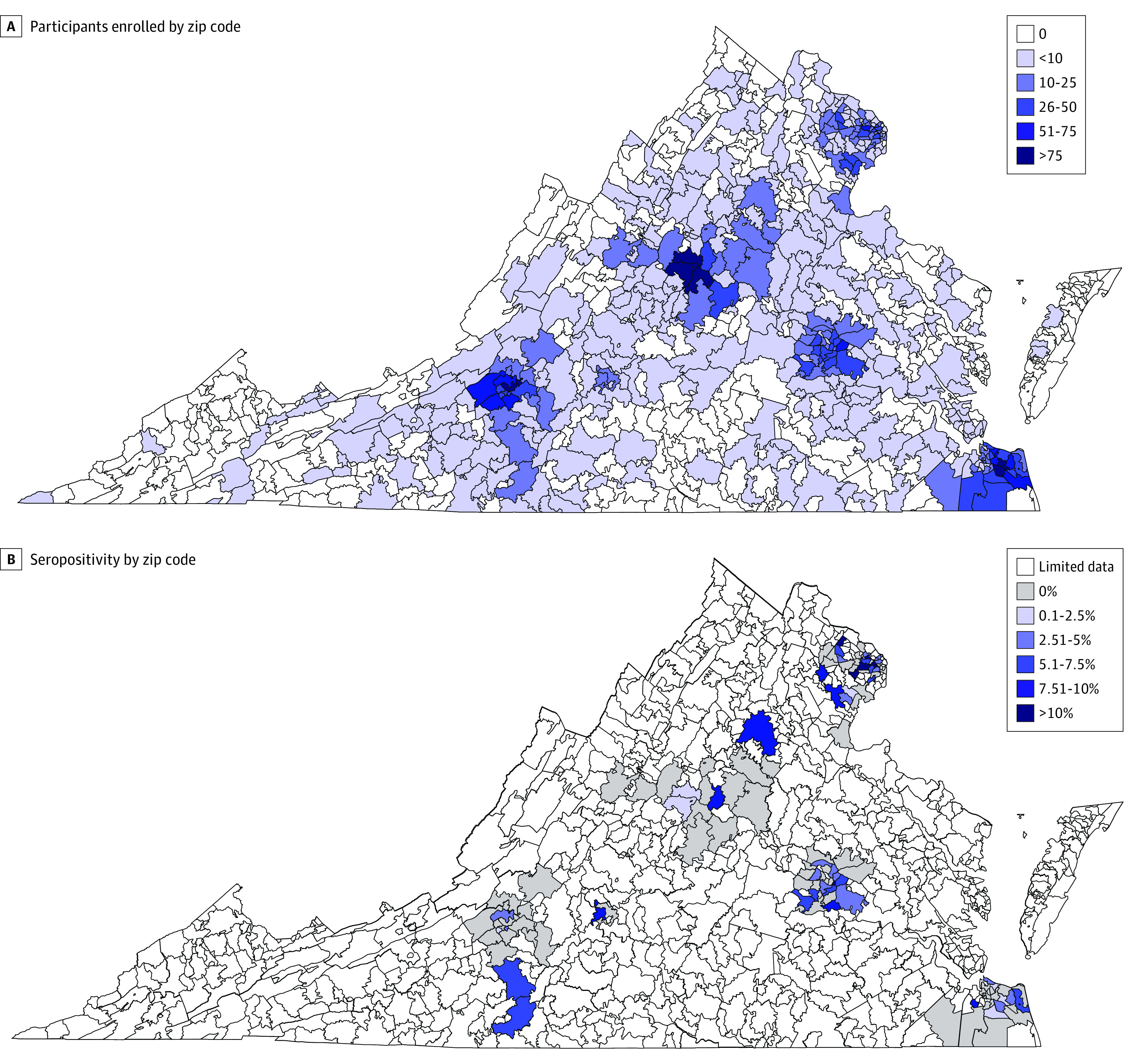
Virginia Coronavirus Seropositivity Maps were created with simplemaps.com. A, Participants enrolled by zip code. Data for any zip code representing a post office were collapsed into the data for the zip code of the post office’s surrounding geographic area. B, Seropositivity by zip code. Only zip codes with at least 10 participants are shown.

The age, race, and ethnic proportions of participants approximated our prespecified enrollment quotas based on the demographic profile of each region. Health insurance, as of January 1, 2020, was captured as a socioeconomic factor; 530 participants (11.3%) had Medicaid coverage, 886 participants (19.0%) had Medicare coverage, 2857 participants (61.1%) had private insurance, 174 participants (3.7%) had military insurance, and 152 participants (3.3%) had no insurance. Most participants (2907 individuals [62.2%]) presented to the clinic for a routine visit for a previous health condition; 1127 participants (24.1%) presented for preventive or well visits, and 478 participants (10.2%) presented for acute illness. Overall, 1854 participants (39.7%) reported having a high-risk health condition, which was defined as diabetes, lung disease (including moderate to severe asthma), severe heart condition, kidney disease, liver disease, or immunocompromise. Most participants (3489 individuals [74.6%]) lived in single-family homes, with a mean (SD) of 0.6 (1.0) children. A total of 2827 participants (60.5%) had not worked outside the home since March 23, 2020, the date of the Virginia stay-at-home order. Having contact with someone with positive test results for COVID-19 infection at or around the time the person was ill was reported by 367 participants (7.9%). A total of 235 participants (5.0%) reported international travel, and 1674 participants (35.8%) reported domestic travel since January 1, 2020. Regarding their level of concern about becoming ill with COVID-19 infection, 3522 participants (75.3%) reported being very or somewhat concerned, and 3726 participants (79.7%) reported being extremely careful about reducing exposure by staying at home, social distancing, washing hands, and wearing cloth face coverings.

We then examined the prevalence of SARS-CoV-2 immunoglobulin G antibodies across the commonwealth and by subgroup (Table 1). Overall, the unadjusted prevalence was 101 of 4675 participants (2.2%). Of 101 participants with seropositivity, 42 participants (41.6%) were Hispanic. Reweighted by region, age, sex, race, ethnicity, and health insurance status to match the census data, the seroprevalence was 2.4% (95% CI, 1.8%-3.1%). When corrected for the 92.7% sensitivity and 99.9% specificity of the assay,^3^ the seroprevalence was 2.5% (95% CI, 1.8%-3.3%). When corrected for a sensitivity of approximately78% (the sensitivity noted in asymptomatic persons^3^), the seroprevalence was 3.0% (95% CI, 1.7%-4.3%).

**Table 1.** SARS-CoV-2 Seroprevalence Across Virginia by Geographic Region and Subgroup

Region or subgroup	Total participants, No. (%)	Participants with seropositivity, No. (%)	Unadjusted prevalence, %	Adjusted prevalence, % (95% CI)
Virginia overall, No.	4675	101	2.2	2.4 (1.8 to 3.1)
Geographic region				
Central	865 (18.5)	20 (19.8)	2.3	2.7 (1.2 to 4.1)
East	976 (20.9)	16 (15.8)	1.6	1.8 (0.3 to 3.4)
North	1001 (21.4)	44 (43.6)	4.4	4.4 (2.8 to 6.1)
Northwest	994 (21.3)	14 (13.9)	1.4	1.1 (0.4 to 1.7)
Southwest	839 (17.9)	7 (6.9)	0.8	0.9 (0.1 to 1.7)
Age range, y				
18-29	735 (15.7)	20 (19.8)	2.7	3.1 (1.5 to 4.7)
30-39	950 (20.3)	21 (20.8)	2.2	2.3 (1.1 to 3.6)
40-49	671 (14.4)	23 (22.8)	3.4	4.4 (1.8 to 7.1)
50-59	933 (20.0)	12 (11.9)	1.3	1.0 (0.2 to 1.7)
60-69	781 (16.7)	13 (12.9)	1.7	1.3 (0.5 to 2.1)
70-79	482 (10.3)	9 (8.9)	1.9	2.1 (0.7 to 3.4)
≥80	123 (2.6)	3 (2.9)	2.4	2.6 (−0.9 to 6.2)
Sex				
Female	3119 (66.7)	74 (73.3)	2.4	2.5 (1.8 to 3.2)
Male	1556 (33.3)	27 (26.7)	1.7	2.4 (1.3 to 3.5)
Race				
White	3098 (66.3)	53 (52.5)	1.7	2.5 (1.6 to 3.3)
Black or African American	942 (20.2)	25 (24.8)	2.7	2.9 (1.4 to 4.4)
Asian	281 (6.0)	3 (2.9)	1.1	1.3 (−0.3 to 3.0)
≥2 Races	76 (1.6)	1 (1.0)	1.3	1.0 (−1.0 to 3.1)
Other race^a^	278 (5.9)	19 (18.8)	6.8	6.3 (3.3 to 9.3)
Ethnicity				
Hispanic	396 (8.5)	42 (41.6)	10.6	10.2 (6.1 to 14.3)
Non-Hispanic	4279 (91.5)	59 (58.4)	1.4	1.6 (1.1-2.2)
Health insurance as of January 1, 2020^b^				
Medicaid^c^	530 (11.3)	9 (8.9)	1.7	3.1 (0.4 to 5.8)
Medicare	886 (19.0)	10 (9.9)	1.1	1.4 (0.3 to 2.4)
Private (employer or individual)	2857 (61.1)	54 (53.5)	1.9	1.8 (1.2 to 2.5)
Military, TRICARE, or Veterans Administration	174 (3.7)	1 (1.0)	0.6	0.7 (−0.7 to 2.0)
None or uninsured	152 (3.3)	13 (12.9)	8.6	5.9 (1.5 to 10.3)
High-risk health condition^d^				
Yes	1854 (39.7)	36 (35.6)	1.9	2.2 (1.1 to 3.4)
No	2786 (59.6)	65 (64.4)	2.3	2.6 (1.8 to 3.4)
Reason for presenting to care^e^				
New or acute illness or injury	478 (10.2)	9 (8.9)	1.9	2.6 (0.6 to 4.5)
Prevention or well visit	1127 (24.1)	24 (23.8)	2.1	2.6 (1.3 to 3.9)
Routine visit for previous condition	2907 (62.2)	65 (64.4)	2.2	2.4 (1.5 to 3.3)
Other or don’t know	160 (3.4)	3 (2.9)	1.9	1.9 (−0.7 to 4.5)

Abbreviation: SARS-CoV-2, severe acute respiratory syndrome coronavirus 2.

^a^ Specific races not queried on survey.

^b^ Health insurance information was missing for 76 individuals.

^c^ Medicaid includes Family Access to Medical Insurance Security (FAMIS), Virginia’s health insurance program for children.

^d^ High-risk health condition was defined as diabetes, lung disease (including moderate to severe asthma), severe heart condition, kidney disease, liver disease, or immunocompromise. Information regarding high-risk health condition was missing for 35 individuals.

^e^ Reason for presenting to care was missing for 3 individuals.

Adjusted seroprevalence varied by geographic area, ranging from 0.9% (95% CI, 0.1%-1.7%) in the southwestern region to 4.4% (95% CI, 2.8%-6.1%) in the northern region. There was substantial heterogeneity in seroprevalence by zip code, ranging from 0% to 20%, often with disparate seroprevalences adjacent to each other (Figure). Seroprevalence was higher than the overall prevalence in Virginia (2.4%; 95% CI, 1.8%-3.1%) among Hispanic individuals (10.2%; 95% CI, 6.1%-14.3%), individuals aged 40 to 49 years (4.4%; 95% CI, 1.8%-7.1%), and individuals who were uninsured (5.9%; 95% CI, 1.5%-10.3%) or insured through Medicaid (3.1%; 95% CI, 0.4%-5.8%). Higher seroprevalence was also observed among participants aged 18 to 29 years (3.1%; 95% CI, 1.5%-4.7%) and 30-39 years (2.3%; 95% CI, 1.1%-3.6%) compared with those aged 50 to 59 years (1.0%; 95% CI, 0.2%-1.7%) and 60 to 69 years (1.3%; 95% CI, 0.5%-2.1%). No change in seropositivity was observed during the time of enrollment between June 1 and August 14, 2020.

We compared the serologic test results with reports of previous COVID-19–like illness or previous COVID-19 test result (ie, viral PCR result). In total, 825 of 4675 participants (17.6%) reported experiencing a COVID-19–like illness (ie, had symptoms such as fever, cough, shortness of breath, chills, muscle pain, sore throat, or new loss of taste or smell) since February 1, 2020. Of those, 518 individuals (62.8%) presented for health care, 66 individuals (8.0%) were hospitalized, and 175 individuals (21.2%) reported receiving a previous test for COVID-19 infection. Among 175 participants with previous symptoms and a previous COVID-19 test, 31 individuals (17.7%) reported receiving a positive result. An additional 649 of 4675 participants (13.9%) received testing for COVID-19 infection for other reasons, of whom 16 individuals (2.5%) reported receiving a positive result.

Among the 47 total individuals who reported a previous positive result for COVID-19 infection, the sensitivity of serology results was 94% (95% CI, 70%-100%). Of the 101 total participants who were seropositive, 50 individuals (49.5%) reported ever having any COVID-19–like illness. Thus, at least 51 individuals (50.5%) with seropositivity were asymptomatic. This estimate assumed that the 19 symptomatic individuals with seropositivity who did not receive PCR testing all had COVID-19 infection; however, it is more likely that only a fraction of these individuals were infected (eg, >18% of individuals with COVID-19–like symptoms who received PCR testing had positive results for COVID-19 infection). Assuming that 3 of 19 individuals (15.8%) who received PCR testing had COVID-19 infection and including the estimated 31 of 175 individuals (17.7%) who were symptomatic and had confirmed positive results for COVID-19 infection on PCR testing, we estimated that 34 of the 101 individuals (33.7%) with seropositivity had confirmed or likely symptomatic infection, rendering 67 individuals (66.3%) with seropositivity associated with asymptomatic infection.

We compared our statewide serologic estimates (seropositivity rate multiplied by population) with the statewide counts of confirmed and probable cases. Given that our serology study’s median enrollment date was July 7, 2020, and given the expected delay between infection and seropositivity, we compared our estimates with the cumulative case counts as of July 1, 2020. As of this date, there were 74 155 individuals with PCR-positive results among 691 698 tests performed, or 849 individuals with PCR-positive results per 100 000 people (PCR results by zip code are available in eFigure 2 in the Supplement).^12^ In addition, we excluded case counts among persons living in long-term care facilities, prisons, or congregate settings to better match the ambulatory population of this study. After these exclusions, we identified 57 680 cases as of July 1, 2020, compared with 162 814 serologically estimated cases (95% CI, 119 256-206 373 cases), or 1864 serologically estimated cases per 100 000 people. Thus, our analysis of serologic data estimated a 2.8-fold increase in total prevalence vs the estimates ascertained by reported case numbers.

We then examined risk factors associated with seropositivity (Table 2). In the univariable analysis, a lower risk of seropositivity was associated with residing in the northwestern or southwestern regions (OR, 0.40 [95% CI, 0.17-0.94] and 0.33 [95% CI, 0.12-0.93]) and being 50 years or older (OR, 0.44; 95% CI, 0.26-0.76). A higher risk of seropositivity was associated with Hispanic ethnicity (OR, 6.85; 95% CI, 3.85-12.16); lack of health insurance (OR, 3.35; 95% CI, 1.41-7.96); living in a multifamily dwelling, apartment, or condominium (OR, 2.88; 95% CI, 1.67-4.98); and having contact with an individual with confirmed COVID-19 infection (OR, 4.26; 95% CI, 2.05-8.88). In the multivariable analysis, residing in the northwestern region (adjusted OR [aOR], 0.38; 95% CI, 0.15-0.98); having Hispanic ethnicity (aOR, 3.56; 95% CI, 1.76-7.21); living in a multifamily residence, apartment, or condominium (aOR, 2.55; 95% CI, 1.25-5.22); and having contact with an individual with confirmed COVID-19 infection (aOR, 4.33; 95% CI, 1.77-10.58) retained statistical significance.

**Table 2.** Risk Factors Associated With Seropositivity^a^

Characteristic	Participants, No. (%) (N = 4675)^b^	OR (95% CI)	
		Univariable model	Multivariable model^c^
Geographic region			
East	976 (20.9)	0.68 (0.24-1.92)	0.92 (0.35-2.40)
North	1001 (21.4)	1.70 (0.85-3.39)	1.30 (0.55-3.11)
Northwest	994 (21.3)	0.40 (0.17-0.94)	0.38 (0.15-0.98)
Southwest	839 (17.9)	0.33 (0.12-0.93)	0.54 (0.18-1.62)
Central	865 (18.5)	1.0 [Reference]	1.0 [Reference]
Age range, y			
≥50	2319 (49.6)	0.44 (0.26-0.76)	0.93 (0.50-1.75)
18-49	2356 (50.4)	1.0 [Reference]	1.0 [Reference]
Sex			
Male	1556 (33.3)	0.96 (0.55-1.67)	NA
Female	3119 (66.7)	1.0 [Reference]	
Race			
Black or African American	942 (20.1)	1.19 (0.64-2.23)	1.19 (0.49-2.86)
Asian	281 (6.0)	0.53 (0.14-1.98)	0.61 (0.15-2.51)
≥2 Races or other race^d^	354 (7.6)	0.80 (0.30-2.15)	0.61 (0.19-2.04)
White	3098 (66.3)	1.0 [Reference]	1.0 [Reference]
Ethnicity			
Hispanic	396 (8.5)	6.85 (3.85-12.16)	3.56 (1.76-7.21)
Non-Hispanic	4279 (91.5)	1.0 [Reference]	1.0 [Reference]
Health insurance as of January 1, 2020			
Medicaid^e^	530 (11.3)	1.72 (0.66-4.50)	1.78 (0.67-4.76)
Medicare	886 (19.0)	0.73 (0.32-1.69)	1.14 (0.43-3.00)
Military, TRICARE, or Veterans Administration	174 (3.7)	0.37 (0.05-2.75)	0.45 (0.06-3.39)
None or uninsured	152 (3.3)	3.35 (1.41-7.96)	2.24 (0.86-5.78)
Private (employer or individual)	2857 (61.1)	1.0 [Reference]	1.0 [Reference]
Reason for visiting clinic			
Prevention or well visit	1127 (24.1)	1.01 (0.40-2.60)	NA
Routine visit for previous condition	2907 (62.2)	0.93 (0.39-2.24)	
Other or don’t know	160 (3.4)	0.75 (0.15-3.66)	
New or acute illness or injury	478 (10.2)	1.0 [Reference]	
High-risk health condition			
Yes	1854 (39.7)	0.85 (0.46-1.57)	NA
No	2786 (59.6)	1.0 [Reference]	
Type of residence			
Other^f^	1186 (25.4)	2.88 (1.67-4.98)	2.55 (1.25-5.22)
Single-family home	3489 (74.6)	1.0 [Reference]	1.0 [Reference]
People in household, median (IQR)			
Adults	2 (0-1.0)	1.15 (1.03-1.29)	1.11 (0.99-1.24)
Children	0 (0-1.0)	1.38 (1.16-1.63)	1.16 (0.92-1.46)
Worked outside the home since March 23, 2020, h/wk			
≤20	482 (10.3)	1.29 (0.63-2.61)	NA
>20	1363 (29.2)	0.82 (0.39-1.75)	
None	2827 (60.5)	1.0 [Reference]	
Worked in health care setting since March 23, 2020^g^			
Yes	719 (15.4)	1.27 (0.51-3.17)	NA
No	1123 (24.0)	1.0 [Reference]	
Work in an essential service^h^			
Yes	1511 (32.3)	0.80 (0.29-2.22)	NA
No	323 (6.9)	1.0 [Reference]	
Known contact with individual with confirmed COVID-19 infection			
Yes	367 (7.9)	4.26 (2.05-8.88)	4.33 (1.77-10.58)
No	4295 (91.9)	1.0 [Reference]	1.0 [Reference]
Travel outside the US since January 1, 2020			
Yes	235 (5.0)	1.41 (0.55-3.62)	NA
No	4436 (94.9)	[Ref]	
Travel outside of Virginia since January 1, 2020			
Yes	1674 (35.8)	0.65 (0.37-1.15)	NA
No	2997 (64.1)	1.0 [Reference]	
Self-reported concern about self or household becoming ill with COVID-19 infection			
Not concerned	1150 (24.6)	1.13 (0.55-2.35)	NA
Somewhat concerned	2023 (43.3)	0.84 (0.45-1.54)	
Very concerned	1499 (32.1)	1.0 [Reference]	
Self-reported carefulness about reducing exposures			
Not careful	43 (0.9)	0.58 (0.11-2.94)	NA
Somewhat careful	903 (19.3)	0.83 (0.42-1.61)	
Extremely careful	3726 (79.7)	1.0 [Reference]	

Abbreviations: COVID-19, coronavirus disease 2019; IQR, interquartile range; NA, not applicable; OR, odds ratio.

^a^ Univariable and multivariable analyses were performed examining geographic region, age, sex, race, ethnicity, health insurance status, reason for presenting to clinic, presence of high-risk health condition, type of dwelling, median number of adults or children in the household, work status, travel status, and self-reported concerns about COVID-19 infection.

^b^ Data are percentages unless otherwise indicated. Data may not always sum to 100% because of rounding, participants who answered “don’t know,” or missing data (which accounted for <1% of data, with the exception of health insurance information, which was missing for 76 participants). Details are available in the eTable in the Supplement.

^c^ Only risk factors with statistically significant ORs (*P* < .05) or ORs less than 0.67 or greater than 1.50 were included in the final multivariable model (n = 4451). NA indicates that the factor was not examined in the multivariable model.

^d^ Specific races not queried on survey.

^e^ Medicaid includes Family Access to Medical Insurance Security (FAMIS), Virginia’s health insurance program for children.

^f^ Multifamily residence, apartment, condominium, long-term care facility or other congregate setting, or other type of residence.

^g^ Only participants who indicated that they worked outside the home (≤20 hours per week or >20 hours per week) were queried about work in a health care setting, which included work in a hospital, physician’s office, outpatient clinic, long-term care facility, or assisted-living facility.

^h^ Only participants who indicated that they worked outside the home (≤20 hours per week or >20 hours per week) were queried about work in an essential service.

## Discussion

This large statewide cross-sectional seroprevalence study was performed to ascertain the extent of previous exposure to SARS-CoV-2 and risk factors associated with COVID-19 infection. Among 4675 demographically representative adults, there was an overall adjusted SARS-CoV-2 seroprevalence of 2.4%. Important risk factors associated with infection were Hispanic ethnicity, residence in a multifamily unit, and contact with an individual with confirmed COVID-19 infection. Seroprevalence was lower in older populations (eg, participants aged 50-69 years vs participants aged 18-29 years) and was higher among residents in the northern region.

This 2.4% seroprevalence is within expectations. In a large seroprevalence study using residual sera samples from individuals in several states, seroprevalence estimates ranged from 1.0% to 6.9%.^4^ Other seroprevalence studies from California, Indiana, and Georgia that were performed during the spring of 2020 have been within this 1.1% to 3.0% range.^5,6,7^ Among frontline health care personnel, seropositivity has been higher, with 1 study reporting seropositivity of 7%.^13^ In contrast, New York City reported the results of more than 1 million antibody tests, finding an estimated 27% seropositivity rate.^14^

Based on the estimated weighted seroprevalence of 2.4%, it is estimated that approximately 2.8-fold more infections occurred than were ascertained by confirmed case counts. This ratio is relatively low compared with the underascertainment estimated by some previous studies, which have ranged from 6-fold to 53-fold more infections than ascertained by confirmed case counts.^4,5^ There are 2 possibilities for the lower underascertainment: (1) the current seroprevalence study captured a relatively lower seroprevalence than that captured by these other studies, and/or (2) the case counts in Virginia are relatively robust and complete. The latter possibility is unlikely because Virginia’s test rate per population (approximately 17 000 of 100 000 people) and our test positivity rate (approximately 6%) were fairly average.^15^ Instead, it is possible that this study’s outpatient participant population could have exhibited a lower seroprevalence than the general population because of higher-than-average health literacy. Notably, however, 39.7% of participants reported having a high-risk health condition, which is similar to reported national estimates.^16^ It also appeared that participants’ level of concern about becoming ill with COVID-19 infection was similar to national norms, with 65% of the US population very or somewhat concerned.^17^ The seroprevalence among individuals aged 18 to 39 years in the study’s outpatient population was low relative to confirmed case counts in the state,^18^ suggesting that this study may underestimate seroprevalence in this group. It is possible that the true seroprevalence in the state is marginally higher than the 2.4% estimate.

### Strengths and Limitations

This study has several strengths. The study had broad geographic reach, which enabled a comparison between regions, and the study had the ability to administer a questionnaire to assess demographic characteristics, risk factors, and previous illness. By enrolling community-dwelling adults receiving outpatient services, the study ensured sufficient enrollment of older adults and individuals with chronic health conditions to assess seroprevalence in this subset of the population who are at high risk of severe illness and hospitalization should they become infected with COVID-19. The highest seropositivity was in the northern region, which is unsurprising given the population density and the location of reported case counts. Notably, seroprevalence was inconsistent, with many zip codes exhibiting 0% seropositivity and others 20% seropositivity, often among adjacent zip codes. The rate of seropositivity was substantially high among Hispanic individuals, at 10.2%. Latinx individuals have been reported to account for a disproportionate number of COVID-19 cases, including in Virginia, in which they account for 33.8%^18^ of cases but only 9% of the general population.^9^ However, it is notable that the seroprevalence in the current study indicated that Hispanic individuals constituted an even higher proportion of seropositive results at 41.6%. Further investigation and preventive strategies targeting Hispanic individuals are needed.

Because the study assessed previous COVID-19 symptoms, it was estimated that at least 50% and more likely 66% of seropositive cases were associated with asymptomatic infection. The asymptomatic rate has been assessed in a number of case series (summarized in a systematic review^19^) and has ranged from 6.3% to 96.0%; however, these case series did not include representative samples from the population. In contrast, community studies from Iceland and Italy estimated the asymptomatic rate to be approximately 40%.^19,20^ These PCR-based studies to define positivity had shorter follow-up periods than the current study. It is important to precisely document the asymptomatic rate because a rate of approximately 66% vs 40% produces large differences in the estimated transmission from asymptomatic individuals.^21^ This study’s data, which indicated a high rate of asymptomatic infection and a higher rate of seropositivity among those who had contact with individuals with confirmed COVID-19 infection, support the testing of asymptomatic individuals who are close contacts as a means of identifying cases and reducing transmission. Although outside the scope of this study, another strength of the study design is that it will be possible to assess this cohort for seroconversion and seroreversion in the future and evaluate whether individuals with seropositivity manifest protection from subsequent COVID-19–like illness.

This study also has several limitations. These limitations include the outpatient participant study design, which may limit generalizability to the general population. In addition, data regarding risk factors and previous testing and illnesses were based on self-reporting and thus subject to participant recall. Notably, however, the most important factors identified (ethnicity, age, and type and geographic location of residence) are not likely to be subject to recall bias. The test sensitivity and specificity of any serologic test is not perfect, and false-positive results increase in a low-prevalence setting. However, the estimates remained similar (and actually increased slightly) after statistical correction for sensitivity and specificity.

## Conclusions

This study estimated that the seroprevalence of SARS-CoV-2 in Virginia was 2.4% as of August 2020. Based on these data, the population in Virginia remains largely immunologically naive to this virus. Herd immunity in Virginia is currently not a plausible means of ending the COVID-19 pandemic.

## Notes

Supplement. **eTable.** Characteristics of Study Population **eFigure 1.** Enrollment by Zip Code **eFigure 2.** Virginia Coronavirus PCR Seropositivity
