# Supplementary material for: Assessment of Seroprevalence of SARS-CoV-2 and Risk Factors Associated With COVID-19 Infection Among Outpatients in Virginia
Source: JAMA Netw Open. 2021 Feb 8;4(2):e2035234. doi: 10.1001/jamanetworkopen.2020.35234 (PMC7871191; doi:10.1001/jamanetworkopen.2020.35234)
Supplement: Supplement. — eTable. Characteristics of Study Population eFigure 1. Enrollment by Zip Code eFigure 2. Virginia Coronavirus PCR Seropositivity [file jamanetwopen-e2035234-s001.pdf]

Supplementary Online Content

Rogawski McQuade ET, Guertin KA, Becker L, et al. Assessment of seroprevalence of SARS-CoV-2 and risk factors associated with COVID-19 infection among outpatients in Virginia. *JAMA Netw Open*. 2021;4(2):e2035234. doi:10.1001/jamanetworkopen.2020.35234

eTable. Characteristics of Study Population

eFigure 1. Enrollment by Zip Code

eFigure 2. Virginia Coronavirus PCR Seropositivity

This supplementary material has been provided by the authors to give readers additional information about their work.

© 2021 Rogawski McQuade ET et al. *JAMA Netw Open*.

eTable. Characteristics of Study Population

	Central (N=865)	East (N=976)	North (N=1001)	Northwest (N=994)	Southwest (N=839)	Overall (N=4675)[a]
**Age**						
18-29	130 (15.0%)	133 (13.6%)	183 (18.3%)	183 (18.4%)	106 (12.6%)	735 (15.7%)
30-39	172 (19.9%)	250 (25.6%)	224 (22.4%)	189 (19.0%)	115 (13.7%)	950 (20.3%)
40-49	117 (13.5%)	119 (12.2%)	193 (19.3%)	132 (13.3%)	110 (13.1%)	671 (14.4%)
50-59	172 (19.9%)	196 (20.1%)	177 (17.7%)	201 (20.2%)	187 (22.3%)	933 (20.0%)
60-69	155 (17.9%)	141 (14.4%)	132 (13.2%)	170 (17.1%)	183 (21.8%)	781 (16.7%)
70-79	99 (11.4%)	100 (10.2%)	73 (7.3%)	96 (9.7%)	114 (13.6%)	482 (10.3%)
80+	20 (2.3%)	37 (3.8%)	19 (1.9%)	23 (2.3%)	24 (2.9%)	123 (2.6%)
**Gender**						
Female	624 (72.1%)	674 (69.1%)	633 (63.2%)	640 (64.4%)	548 (65.3%)	3119 (66.7%)
**Race**						
White	486 (56.2%)	557 (57.1%)	576 (57.5%)	793 (79.8%)	686 (81.8%)	3098 (66.3%)
African American	300 (34.7%)	298 (30.5%)	128 (12.8%)	114 (11.5%)	102 (12.2%)	942 (20.1%)
Asian	34 (3.9%)	48 (4.9%)	163 (16.3%)	26 (2.6%)	10 (1.2%)	281 (6.0%)
Other race	26 (3.0%)	59 (6.0%)	120 (12.0%)	50 (5.0%)	23 (2.7%)	278 (5.9%)
Two or more races	19 (2.2%)	14 (1.4%)	14 (1.4%)	11 (1.1%)	18 (2.1%)	76 (1.6%)
**Ethnicity**						
Hispanic	40 (4.6%)	69 (7.1%)	179 (17.9%)	81 (8.1%)	27 (3.2%)	396 (8.5%)
non-Hispanic	825 (95.4%)	907 (92.9%)	822 (82.1%)	913 (91.9%)	812 (96.8%)	4279 (91.5%)
**Insurance status as of Jan 1, 2020**						
Medicaid [b]	82 (9.5%)	57 (5.8%)	65 (6.5%)	192 (19.3%)	134 (16.0%)	530 (11.3%)
Medicare	181 (20.9%)	184 (18.9%)	127 (12.7%)	162 (16.3%)	232 (27.7%)	886 (19.0%)
Private (employer or individual)	551 (63.7%)	631 (64.7%)	690 (68.9%)	557 (56.0%)	428 (51.0%)	2857 (61.1%)
Military [c]	11 (1.3%)	88 (9.0%)	42 (4.2%)	21 (2.1%)	12 (1.4%)	174 (3.7%)
None or uninsured	29 (3.4%)	12 (1.2%)	34 (3.4%)	52 (5.2%)	25 (3.0%)	152 (3.3%)
**Reason for presenting to clinic** [d]						
New or acute illness or injury	55 (6.4%)	95 (9.7%)	60 (6.0%)	166 (16.7%)	102 (12.2%)	478 (10.2%)

© 2021 Rogawski McQuade ET et al. *JAMA Netw Open*.

	Central (N=865)	East (N=976)	North (N=1001)	Northwest (N=994)	Southwest (N=839)	Overall (N=4675)[a]
Prevention or Well visit	120 (13.9%)	275 (28.2%)	366 (36.6%)	213 (21.4%)	153 (18.2%)	1127 (24.1%)
Routine visit for a prior condition	664 (76.8%)	605 (62.0%)	533 (53.2%)	596 (60.0%)	509 (60.7%)	2907 (62.2%)
Other or don't know	25 (2.9%)	1 (0.1%)	40 (4.0%)	19 (1.9%)	75 (8.9%)	160 (3.4%)
**High risk health condition** [e]						
Yes	379 (43.8%)	458 (46.9%)	305 (30.5%)	384 (38.6%)	328 (39.1%)	1854 (39.7%)
No	478 (55.3%)	517 (53.0%)	688 (68.7%)	603 (60.7%)	500 (59.6%)	2786 (59.6%)
**Type of dwelling**						
Single family	669 (77.3%)	773 (79.2%)	645 (64.4%)	726 (73.0%)	676 (80.6%)	3489 (74.6%)
Multi-family / Apartment / Condo building	189 (21.8%)	201 (20.6%)	346 (34.6%)	250 (25.2%)	158 (18.8%)	1144 (24.5%)
Long-term care facility or other congregate setting	4 (0.5%)	2 (0.2%)	2 (0.2%)	6 (0.6%)	3 (0.4%)	17 (0.4%)
**Number of adults in household**						
Mean (SD)	2.1 (± 1.3)	2.2 (± 1.1)	2.4 (± 1.2)	2.2 (± 1.6)	2.1 (± 0.99)	2.2 (± 1.3)
**Number of children in household**						
Mean (SD)	0.59 (± 1.0)	0.64 (± 1.0)	0.77 (± 1.1)	0.56 (± 0.99)	0.47 (± 0.87)	0.61 (± 1.0)
**Worked outside the home since March 23, 2020 (date of stay at home order)**						
Not at all	507 (58.6%)	528 (54.1%)	676 (67.5%)	593 (59.7%)	523 (62.3%)	2827 (60.5%)
Up to 20 hours per week	103 (11.9%)	97 (9.9%)	105 (10.5%)	114 (11.5%)	63 (7.5%)	482 (10.3%)
More than 20 hours per week	254 (29.4%)	351 (36.0%)	218 (21.8%)	287 (28.9%)	253 (30.2%)	1363 (29.2%)

© 2021 Rogawski McQuade ET et al. *JAMA Netw Open*.

	Central (N=865)	East (N=976)	North (N=1001)	Northwest (N=994)	Southwest (N=839)	Overall (N=4675)[a]
**Work in a hospital, doctor's office, outpatient clinic, long-term care facility, or assisted living facility**						
Yes	164 (19.0%)	145 (14.9%)	130 (13.0%)	190 (19.1%)	90 (10.7%)	719 (15.4%)
No	192 (22.2%)	303 (31.0%)	192 (19.2%)	210 (21.1%)	226 (26.9%)	1123 (24.0%)
**Work in an essential service**						
Yes	283 (32.7%)	372 (38.1%)	266 (26.6%)	320 (32.2%)	270 (32.2%)	1511 (32.3%)
No	72 (8.3%)	73 (7.5%)	56 (5.6%)	76 (7.6%)	46 (5.5%)	323 (6.9%)
**Contact with COVID+ individual**						
Yes	81 (9.4%)	72 (7.4%)	113 (11.3%)	59 (5.9%)	42 (5.0%)	367 (7.9%)
No/don't know	783 (90.5%)	904 (92.6%)	886 (88.5%)	935 (94.0%)	797 (95.0%)	4295 (92.1%)
**Travel outside the country since Jan 1, 2020**						
Yes	36 (4.2%)	33 (3.4%)	88 (8.8%)	53 (5.3%)	25 (3.0%)	235 (5.0%)
No	827 (95.6%)	943 (96.6%)	911 (91.0%)	941 (94.7%)	814 (97.0%)	4436 (94.9%)
**Travel outside the state since Jan 1, 2020**						
Yes	278 (32.1%)	294 (30.1%)	507 (50.6%)	322 (32.4%)	273 (32.5%)	1674 (35.8%)
No	585 (67.6%)	682 (69.9%)	492 (49.2%)	672 (67.6%)	566 (67.5%)	2997 (64.1%)
**Self-reported concern about you or your household getting sick with COVID-19**						
Very concerned	306 (35.4%)	370 (37.9%)	309 (30.9%)	278 (28.0%)	236 (28.1%)	1499 (32.1%)
Somewhat concerned	368 (42.5%)	368 (37.7%)	457 (45.7%)	457 (46.0%)	373 (44.5%)	2023 (43.3%)
Not concerned	190 (22.0%)	238 (24.4%)	233 (23.3%)	259 (26.1%)	230 (27.4%)	1150 (24.6%)

© 2021 Rogawski McQuade ET et al. *JAMA Netw Open*.

	Central (N=865)	East (N=976)	North (N=1001)	Northwest (N=994)	Southwest (N=839)	Overall (N=4675)[a]
**Self-reported carefulness about reducing exposures**						
Extremely careful	748 (86.5%)	786 (80.5%)	811 (81.0%)	746 (75.1%)	635 (75.7%)	3726 (79.7%)
Somewhat careful	114 (13.2%)	177 (18.1%)	184 (18.4%)	234 (23.5%)	194 (23.1%)	903 (19.3%)
Not careful	2 (0.2%)	13 (1.3%)	4 (0.4%)	14 (1.4%)	10 (1.2%)	43 (0.9%)

[a] Data may not always sum to 100% if participants did not know or data was missing (< 1%); Data were missing for health insurance (n=76), reason for presenting to clinic (n=3), high risk health condition (n=35), number of adults in household (n=4), number of children in household (n=5), work outside the home (n=3), work in a hospital, doctor's office, outpatient clinic, long-term care facility, or assisted living facility (n=3), work in an essential service (n=11), contact with a COVID+ individual (n=3), travel outside the country (n=4), travel outside the state (n=4), self-reported concern (n=3), self-reported carefulness (n=3)

[b] Medicaid includes FAMIS, Virginia's health insurance program for children

[c] includes Tricare or Veterans Administration

[d] no patients were presenting for acute COVID-19 illness; reason for presenting to clinic missing for n=3 participants

[e] diabetes, lung disease (including moderate to severe asthma), a severe heart condition, kidney disease, liver disease, or immunocompromise

© 2021 Rogawski McQuade ET et al. *JAMA Netw Open*.

eFigure 1. Enrollment by Zip Code

(A) Enrollment rate by zip code. The zip code is colored based on the number of individuals enrolled per 100,000 population in the zip code.

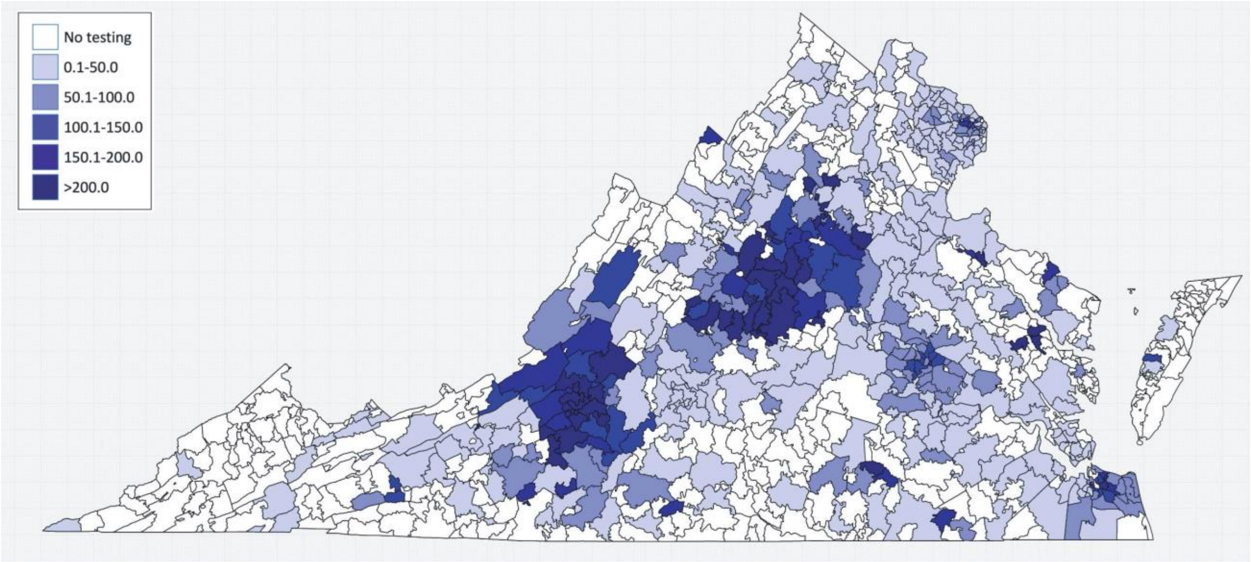

(B) Population of each zip code in Virginia. Data from https://www.virginia-demographics.com/zip_codes_by_population. Maps created with simplemaps.com.

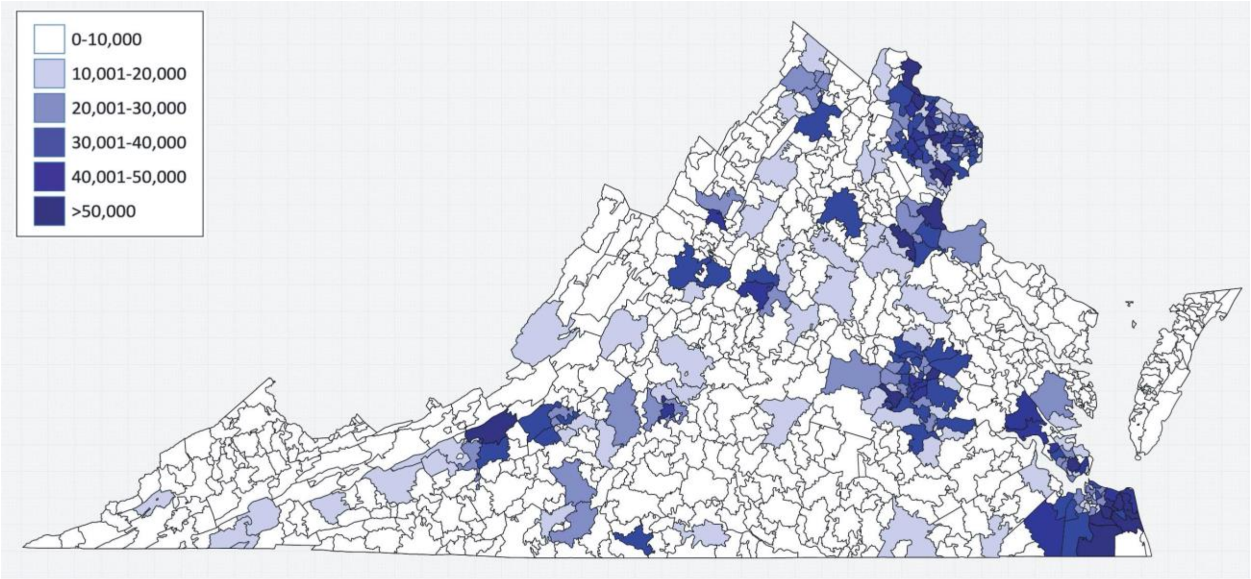

© 2021 Rogawski McQuade ET et al. *JAMA Netw Open*.

eFigure 2. Virginia Coronavirus PCR Seropositivity

Rate of positive SARS-CoV2 PCR results as of July 1, 2020 per 100,000 population by zip code. Data from https://data.virginia.gov/Government/VDH-COVID-19-PublicUseDataset-ZIPCode/8bkr-zfqv Maps created with simplemaps.com.

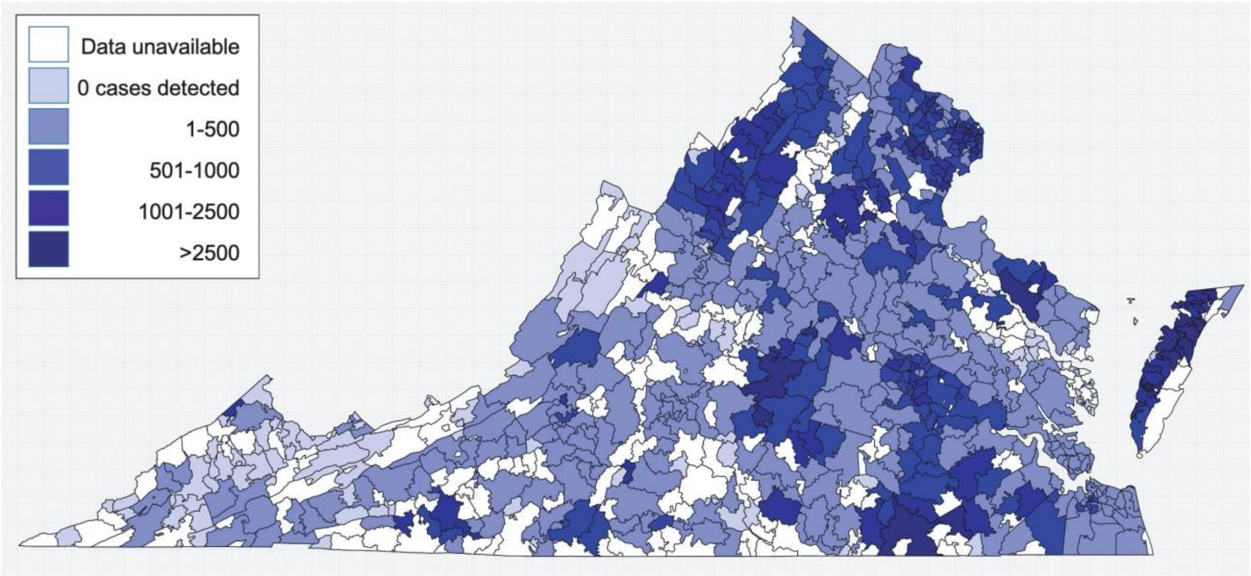

© 2021 Rogawski McQuade ET et al. *JAMA Netw Open*.
